# Modifying meiotic recombination by targeting chromatin regulators to crossover hotspots in *Arabidopsis*

**DOI:** 10.1126/sciadv.aeb2890

**Published:** 2026-03-13

**Authors:** Maja Szymanska-Lejman, Wojciech Dziegielewski, Anna Wilhelm, Karolina Hus, Piotr A. Ziolkowski

**Affiliations:** ^1^ Laboratory of Genome Biology, Institute of Molecular Biology and Biotechnology, Adam Mickiewicz University, Poznan, Poland.; ^2^ Institute of Bioorganic Chemistry, Polish Academy of Sciences, Poznan, Poland.

## Abstract

The impact of specific chromatin modifications on meiotic crossover frequency has typically been inferred from correlative studies, leaving the question of causality unresolved. To directly test this, we used a catalytically inactive CRISPR-associated protein 9 (dCas9)–based system to recruit the histone demethylase JMJ14 to defined genomic loci. Recruitment of JMJ14 led to a reduction in local histone H3 lysine 4 trimethylation (H3K4me3) levels and a decrease in crossover frequency within the targeted interval. This was accompanied by reduced expression of a long noncoding RNA (lncRNA) at the hotspot and altered crossover topology. Suppressed recombination was also observed at neighboring, untargeted hotspots. In contrast, targeting the transcriptional activator VP64 to the same region increased lncRNA expression, elevated crossover frequency, and raised H3K4me3 levels. Together, these findings establish a causal link between H3K4me3, transcription, and local crossover activity, demonstrating that H3K4me3 levels are closely associated with both transcriptional output and recombination frequency at specific genomic loci.

## Teaser

Targeting chromatin modifiers to DNA hotspots reveals causal link between histone marks, transcription, and meiotic recombination.

## INTRODUCTION

Meiotic crossovers represent the most powerful tool available to plant breeders, enabling the combination of beneficial genetic variation to develop cultivars with improved agronomic traits. Typically, however, breeders are interested in transferring only specific loci associated with desirable traits between parental genotypes. This is particularly important for the introgression of disease resistance genes, which are often tightly linked to unfavorable alleles. Unfortunately, no current technology allows for the targeted induction of recombination at specific genomic locations. As a result, breeding programs rely on screening large plant populations, making the process both time consuming and resource intensive.

Meiotic recombination is initiated through the formation of programmed DNA double-strand breaks (DSBs), catalyzed by the evolutionarily conserved transesterase SPO11, in conjunction with the meiotic topoisomerase VIB (MTOPVIB) (*1*–*6*). In most eukaryotes, including plants, only a small fraction of meiotic DSBs is resolved into crossovers, while most are repaired through alternative noncrossover pathways. For example, in *Arabidopsis thaliana*, out of approximately 200 DSBs formed during a single meiotic division, no more than 10 are repaired as crossovers (*7*, *8*). It is possible that this large “excess” of DSBs observed in plants may underlie the ineffectiveness of DSB induction–based strategies to enhance crossover formation at specific loci, unlike in yeast (*9*).

The distribution of crossovers along chromosomes is nonuniform but instead occurs preferentially at specific regions known as recombination hotspots, where the crossover frequency substantially exceeds the genome-wide average (*10*–*13*). In the majority of mammals such as mice and humans, the location of most DSB hotspots is determined by PRDM9, a histone methyltransferase containing a zinc finger DNA binding domain that recognizes specific sequence motifs (*14*–*16*). PRDM9 directs the deposition of histone modifications, including trimethylation of histone H3 at lysines 4 (H3K4me3) and 36 (H3K36me3), which in turn facilitate the recruitment of the DSB machinery (*14*, *16*). Plants lack PRDM9 and therefore use alternative mechanisms to define recombination hotspots. One of the factors stimulating DSB formation in plants is chromatin accessibility as most of the recombination hotspots are observed in low nucleosome density regions, which correspond to gene promoters and 3′ ends of the genes (*13*, *17*–*19*). This coincides with deposition of H2A.Z histone variant at transcription start sites (TSS) and transcription termination sites. Furthermore, several studies have implicated specific histone marks in either promoting or repressing meiotic recombination. For instance, euchromatic features such as histone H3 lysine 4 dimethylation and trimethylation (H3K4me2 and H3K4me3) as well as H3K9 acetylation have been associated with crossover hotspots in multiple species, while heterochromatic marks such as H3K9me2, H3K27me3, or H2A.W histone variant are generally enriched in recombination-poor regions (*20*–*24*). However, the causal relationship between these chromatin states and recombination outcomes remains unclear, particularly in plants, in which direct experimental evidence is limited. Consequently, understanding whether local manipulation of chromatin marks can modulate crossover activity is a critical step toward enabling targeted recombination in plant genomes.

In this study, we used a CRISPR–dead Cas9 (dCas9) platform in *A. thaliana* to test the influence of two specific chromatin effectors, histone demethylase JUMONJI 14 (JMJ14) and transcription activator VP64, on meiotic recombination frequency (RF) in hotspots within defined pericentromeric and interstitial intervals. By leveraging dCas9-mediated recruitment of these factors to fluorescently tagged crossover hotspots, we established a versatile and quantitative assay for probing recombination outcomes. Our findings highlight a critical role for H3K4me3 in promoting crossover activity, as demonstrated by the suppressive effect of the H3K4 demethylase JMJ14. In contrast, enhancing transcription through VP64 activation significantly elevated crossover rates, underscoring a tight link between transcriptional activity and hotspot function.

## RESULTS

### A system for testing the effects of chromatin modification on crossover formation

**Fig. 1.**
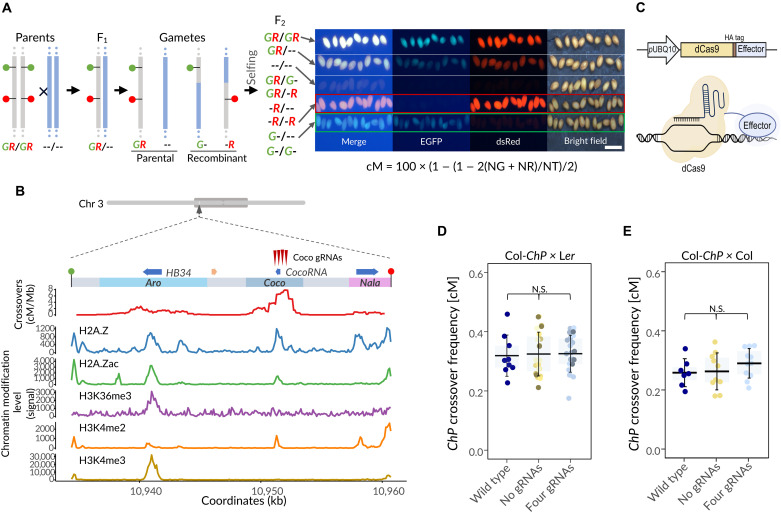
System for studying the impact of chromatin modifications on crossover frequency. (**A**) Measurement of crossover frequency (in centimorgans) using ESILs with segregating fluorescent reporters. Vertical gray and blue lines represent homologous chromosomes from Col (ESIL) and L*er*, respectively; green and red markers indicate fluorescent reporters. All possible F2 genotypes are shown. Representative images of seeds from the Col-ChP × L*er* cross are shown on the right, together with the formula used for centimorgan calculation; NG, green-only seeds; NR, red-only seeds; NT, total seed number. (**B**) Chromosomal location, genes, and chromatin landscape of the *ChP* interval. The top shows the position of *ChP* on chromosome 3, with the pericentromeric region indicated by a dark gray oval. A zoomed-in view highlights the *Aro*, *Coco*, and *Nala* crossover hotspots. Blue arrows indicate genes, and an orange arrow marks a pseudogene. Maroon arrowheads mark gRNAs used in this study. Wild-type crossover frequency (centimorgan per megabase) is plotted as a moving average (1-kb window, 100-bp step). Chromatin modification levels are shown in 100-bp windows. Crossover data from (*26*), H2A.Z and H2A.Zac levels from (*46*), H3K36me3 from (*51*), H3K4me2 from (*52*), and H3K4me3 from (*53*). (**C**) Schematic of the constructs used for dCas9-effector expression and the mode of action of the resulting fusion protein. (**D** and **E**) Crossover frequency in crosses involving transformants expressing dCas9 either without gRNAs or with four gRNAs targeting the *Coco* hotspot. In the boxplots, the center line represents the mean, the boxes indicate the 25th and 75th percentiles, and the whiskers show SD. Each data point corresponds to a crossover frequency measurement from a single cross. At least three independent transformants were analyzed for each condition. (D) Col-*ChP* × L*er* crosses and (E) Col-*ChP* × Col crosses. N.S., not significant.

In plants, recombination hotspots are relatively numerous and tend to form discrete local peaks that are frequently located near transcriptionally active genes, particularly in promoter regions and adjacent 5′ or 3′ untranslated regions (UTRs) (*8*, *13*, *17*–*19*, *25*). As a result, determining the direct impact of chromatin state on the recombination activity of hotspots requires a system capable of measuring crossover frequency within short chromosomal regions containing as few hotspots as possible. We recently developed a system based on extremely short chromosomal intervals (ESILs), which are defined by fluorescent reporters expressed in seeds (*26*). By scoring the segregation of these reporters during meiosis, it is possible to precisely measure crossover frequency within the interval (Fig. 1A). Since ESILs span only a few dozen kilobases and contain just a few hotspots, this system is ideal for directly studying how local changes in chromatin state affect crossover frequency.

To test the impact of local chromatin modifications on meiotic recombination, we selected the *Chili Pepper* (*ChP*) interval, which contains three distinct crossover hotspots (Fig. 1B). To locally modify chromatin within these hotspots, we used a system based on a catalytically inactive form of the Cas9 nuclease (dCas9). In this system, dCas9 is fused to a selected effector via translational fusion. The recruitment of dCas9-effectors to specific genomic loci is guided by CRISPR guide RNAs (gRNAs), expressed under *U3* or *U6* promoters (Fig. 1C).

Since dCas9 itself is a large protein (~156 kDa), we considered the possibility that its targeting to a hotspot could cause steric hindrance, limiting access of recombination proteins and thereby reducing crossover frequency. To test this, we directed dCas9 to the strongest hotspot within the *ChP* interval, *Coco*, using four gRNAs complementary to the peak recombination site within the hotspot (Fig. 1B). As a control, we transformed plants with the same dCas9 construct lacking gRNAs. Transformants generated in the Col-*ChP* background were crossed with wild-type L*er* plants, and crossover frequency was measured (fig. S1). The results showed that dCas9 recruitment to *Coco* in the absence of an effector had no effect on crossover formation at the *ChP* interval (Fig. 1D). Similarly, no change was observed when T_1_ lines expressing gRNAs targeting the *Coco* hotspot were crossed to Col (Fig. 1E). These results indicate that dCas9 targeting does not interfere with recombination activity within hotspots, suggesting that steric hindrance is not a limiting factor.

### Recruitment of JMJ14 to a crossover hotspot reduces local crossover frequency

**Fig. 2.**
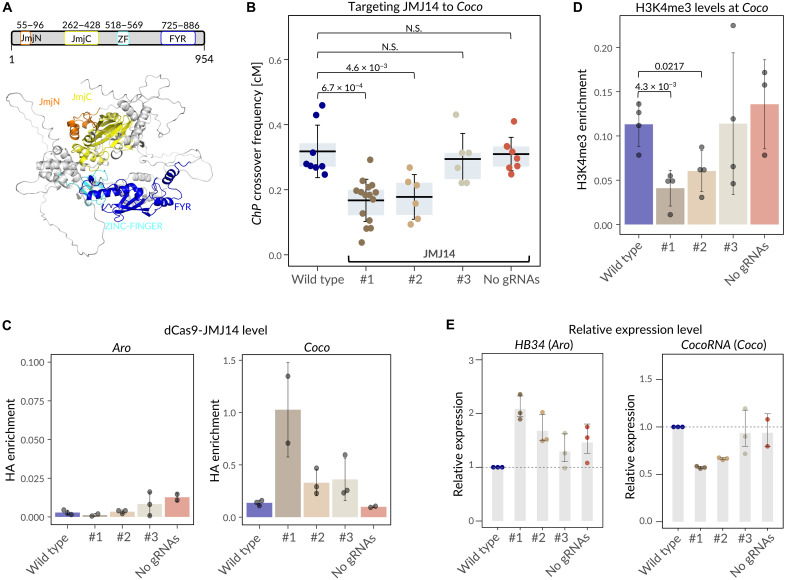
Targeted JMJ14 locally reduces crossover frequency and gene expression, associated with decreased H3K4me3 levels. (**A**) Domain architecture and structure of JMJ14. The top illustrates the domain organization, highlighting JmjN (Jumonji N-terminal), JmjC (Jumonji C-terminal), ZF (Zinc Finger), and FYR (phenylalanine-tyrosine–rich) domains. The previously published JMJ14 crystal structure (Protein Data Bank: 5YKO; UniProt: Q8GUI6) (*54*) is shown with domains highlighted in PyMOL. The JmjN and JmjC domains form the catalytic core responsible for histone demethylation. The ZF domain is implicated in DNA binding, while the FYR domain is likely involved in protein-protein interactions. (**B**) Crossover frequency in the *ChP* interval for crosses with three transgenic lines carrying dCas9-JMJ14 targeted to the *Coco* hotspot (#1 to #3). Controls include wild-type Col-*ChP* × L*er* and lines expressing dCas9-JMJ14 without gRNAs. In the boxplot, the center line indicates the mean, the upper and lower bounds represent the 75th and 25th percentiles, and error bars show the SD. Each data point represents crossover frequency for a single cross. Welch’s test was used to determine statistical significance. (**C**) dCas9-JMJ14 enrichment at the *Aro* and *Coco* hotspots in the crosses used in (B), measured by ChIP-qPCR. Bars represent mean values of three biological replicates (dots), with error bars indicating SD. (**D**) Changes in H3K4me3 enrichment at the *Coco* hotspot in the crosses used in (B), measured by ChIP-qPCR. Bars represent mean values of three to four biological replicates (dots), with error bars indicating SD. Statistical significance was assessed using *t* test. Only significant values are shown. (**E**) Relative expression of the *HB34* gene located within the *Aro* hotspot and the *CocoRNA* gene within the *Coco* hotspot in the crosses used in (B), measured by RT-qPCR. Bars represent mean values of three biological replicates (dots), with error bars indicating SD.

In many eukaryotes, H3K4 di/trimethylation (H3K4me2/3) positively correlates with crossover frequency and is enriched at recombination hotspots (*13*, *20*, *21*, *27*). Therefore, we aimed to determine whether removing these modifications from nucleosomes within the *Coco* hotspot would reduce recombination activity (see Fig. 1B for H3K4me2/3 profile over *Coco* hotspot). We used JMJ14, an enzyme capable of demethylating H3K4me3 and, to a lesser extent, H3K4me2 in vivo (*28*, *29*). To this end, we generated a genetic construct in which full coding sequence of JMJ14 was fused to dCas9 (Fig. 2A and fig. S1). Targeting to the *Coco* hotspot was achieved using a set of four gRNAs previously validated in the steric hindrance assay. As a control, we used constructs expressing functional dCas9-JMJ14 but lacking gRNAs, thereby preventing recruitment to the target hotspot. The genetic constructs were introduced into Col-*ChP* plants and crossed with the nonfluorescent accession, L*er*, to assess crossover frequency by fluorescent seed segregation (fig. S1).

Targeting dCas9-JMJ14 to *Coco* led to a significant decrease in crossover frequency within the *ChP* interval in two of three independent T_1_ lines crossed to the nonfluorescent accession L*er* (Fig. 2B; *P* < 4.6 × 10^−3^, Welch test), while no change was observed in the third. Notably, this effect was absent in control lines lacking gRNAs, in which JMJ14 was not recruited to *Coco* (Fig. 2B).

### Targeted JMJ14 reduces local H3K4me3 levels, leading to both reduced crossover activity and transcriptional repression

To understand the mechanism underlying the observed recombination suppression, we selected the same three T_1_ lines, previously analyzed for crossover frequency, and assessed dCas9-JMJ14 occupancy at the *Coco* and *Aro* hotspots (Fig. 2, B and C). One control line lacking gRNAs was also included. Chromatin immunoprecipitation–quantitative polymerase chain reaction (ChIP-qPCR) analysis, performed using an antibody against the hemagglutinin (HA) tag that is fused to the C terminus of dCas9, confirmed dCas9 enrichment at the *Coco* hotspot in gRNA-targeted lines, relative to wild-type plants. No enrichment was detected in the line without gRNAs (Fig. 2C). dCas9 was also absent at the *Aro* hotspot, which was not targeted by gRNAs (Fig. 2C). These results confirm the efficiency and specificity of dCas9-JMJ14 binding to chromatin regions defined by gRNA sequences.

Next, we examined H3K4me3 enrichment in these lines at the targeted hotspot (Fig. 2D). As expected, H3K4me3 levels were significantly reduced to 0.25 to 0.5 of wild-type levels in two lines exhibiting a decrease in *ChP* crossover frequency (*P* < 0.0217; *t* test). However, in the third line, where dCas9-JMJ14 was detected but no significant change in crossover frequency was observed, H3K4me3 levels remained unchanged (Fig. 2, B to D). In contrast, in the line lacking gRNAs, H3K4me3 methylation was higher than in the wild type. These findings clearly support the conclusion that the reduction in H3K4 trimethylation directly affects hotspot recombination activity.

Similar to most crossover hotspots in *Arabidopsis*, the *Aro* and *Coco* hotspots overlap with actively transcribed genes: *HB34* (AT3G28920) and AT3G05605, respectively (Fig. 1B). AT3G05605 encodes an uncharacterized long noncoding RNA (lncRNA), which we named *CocoRNA*. Since decreased H3K4me3 levels often correlate with transcriptional repression, we investigated whether *CocoRNA* expression was reduced in dCas9-JMJ14 lines using reverse transcription (RT)–qPCR. *CocoRNA* levels were significantly lower than in wild-type plants, correlating with the reduction in *ChP* crossover frequency (Fig. 2E; *P* < 1.1 × 10^−6^, *t* test). However, no changes in *CocoRNA* expression were observed in the line that did not show a crossover frequency reduction or in the line without gRNAs (Fig. 2E). *HB34* expression showed the opposite trend, increasing up to 2.1-fold in lines with reduced crossover frequency compared to wild-type plants, suggesting JMJ14-dependent coregulation of these two neighboring genes (Fig. 2E). Together, these results demonstrate that targeting the histone demethylase altered the chromatin state within the *Coco* hotspot, leading to both reduced recombination activity and transcriptional repression of the hotspot-associated gene.

### JMJ14 targeting alters crossover distribution within and between the three hotspots in the *ChP* interval

**Fig. 3.**
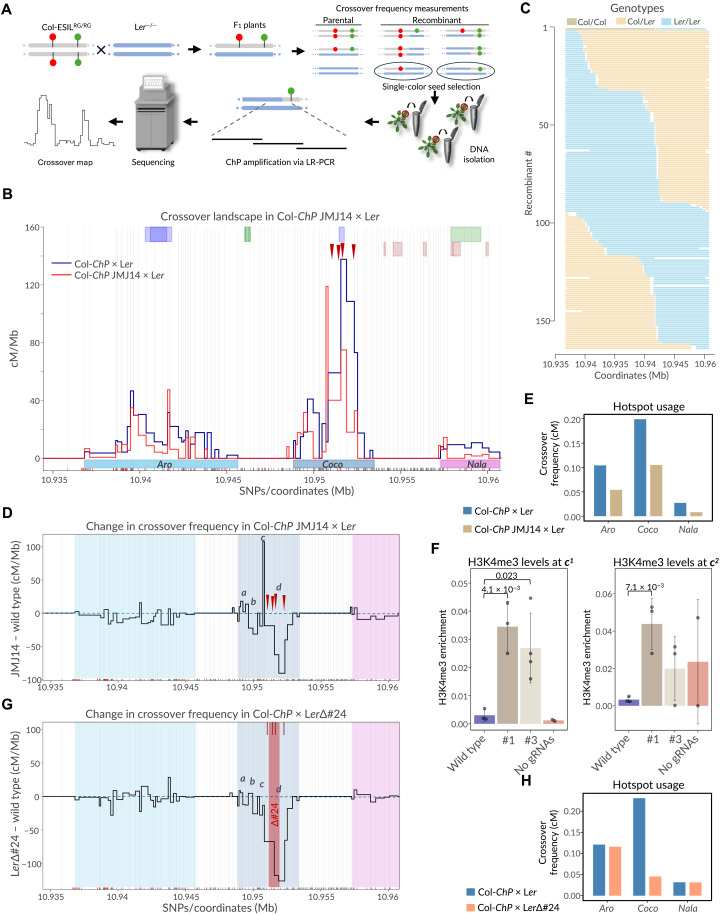
JMJ14 suppresses crossovers at the manipulated hotspot and reshapes local recombination landscape. (**A**) Schematic of seed-based crossover mapping. ESIL × L*er* crosses produce F_1_ plants; crossover frequency within a reporter-marked interval is inferred from fluorescent seed segregation, and single-color seeds are used to map crossover breakpoints by LR-PCR and sequencing. (**B**) Crossover landscape within *ChP* for the Col-*ChP* JMJ14 × L*er* (red; *N* = 164) and wild type (Col-*ChP* × L*er*, blue; *N* = 243). Crossover frequencies are normalized to total *ChP* crossovers. Col/L*er* SNPs used for breakpoint mapping are indicated along the *x* axis; genes (green/violet) and transposons (orange) are shown above. The *Aro*, *Coco*, and *Nala* hotspots are highlighted. gRNAs target sites are indicated by arrowheads. Wild-type data from (*26*). (**C**) Genotypes of individual recombinants from the Col-*ChP* JMJ14 × L*er* cross, shown as color-coded haplotypes across *ChP*. (**D**) Changes in crossover frequency across *ChP* upon JMJ14 targeting to *Coco*, plotted as differences from wild type. Hotspots and gRNA target sites are indicated. Four sectors within *Coco* (*a* to *d*) showing differential crossover remodeling are labeled. (**E**) Hotspot usage for Col-ChP JMJ14 × L*er* versus Col-*ChP* × L*er*. (**F**) H3K4me3 enrichment in the *c* sector of the *Coco* hotspot [see (D)] in the crosses analyzed in Fig. 2B, measured by ChIP-qPCR at two amplicons (*c*^1^ and *c*^2^). Cross #1 was used for seed typing in (B) to (D). Bars represent means of two to four biological replicates (dots); error bars indicate SD. Only significant values (*t* test) are shown. (**G**) Same as in (D), but for Col-*ChP* × L*er*Δ#24 cross [data from (*26*)]. The Δ#24 deletion in *Coco* is indicated. gRNA positions used for JMJ14 targeting are shown as burgundy lines. (**H**) Hotspot usage for Col-*ChP* × L*er*Δ#24 versus Col-*ChP* × L*er*.

To precisely examine changes in the distribution of crossover events within the *ChP* interval in plants with targeted dCas9-JMJ14, we used our recently developed seed typing technique (*26*). Seed typing relies on deep sequencing of recombinant seeds selected from crosses with ESILs. A cross between a Col-*ChP* line carrying dCas9-targeted JMJ14 and a L*er* background generated a Col/L*er* hybrid, which, upon self-pollination, produced seeds segregating for enhanced green fluorescent protein (eGFP) and dsRed reporters. Recombinants were manually selected, and the crossover-containing interval was amplified using high-fidelity long-range PCR (LR-PCR) with overlapping amplicons. The resulting PCR products were used to construct Illumina-compatible libraries for each recombinant, followed by sequencing at ~1500× coverage. Crossover sites were identified on the basis of single-nucleotide polymorphisms (SNPs) distinguishing Col from L*er*. By combining crossover positions from multiple recombinants, we achieved high-resolution mapping of crossover distribution (Fig. 3A).

Comparison of crossover distribution in lines with dCas9-JMJ14 targeted to the *Coco* hotspot revealed substantial differences from the wild type (Fig. 3, B and C). We observed changes in the recombination topography of the *Coco* hotspot, reflected in a markedly altered crossover profile. Specifically, within the region targeted by JMJ14, there was a clear reduction in crossover frequency. However, immediately downstream of this region—still within the *Coco* interval—a short sector displayed an increase in crossover frequency.

To better characterize these topological differences, we performed a differential analysis in which the crossover distribution was normalized to that of the wild type (Fig. 3D). This analysis revealed that the reduction in crossover frequency occurred in two sectors (*b* and *d* in Fig. 3D), separated by a narrow sector (*c*) showing increased crossover frequency. In addition, a rise in crossover frequency was observed in the distal left-hand sector (*a*). A reduction in crossover frequency was also apparent across the other two hotspots, *Aro* and *Nala* (Fig. 3D), which is further reflected in the hotspot usage analysis (Fig. 3E).

We next asked whether the local increase in crossover frequency in sectors *a* and *c* of the *Coco* hotspot is also associated with changes in chromatin state. To address this, we measured H3K4me3 levels at one site in sector *a* and at two sites in sector *c* (*c*^1^ and *c*^2^) by designing qPCR amplicons covering nucleosomes located within these sectors. ChIP-qPCR was performed using progeny from cross #1, which showed the strongest targeting effect (Fig. 2B) and was used for seed typing and crossover mapping (Fig. 3, A to E), as well as from line #3, which displayed only a modest reduction in crossover frequency. At the *c*^1^ and *c*^2^ amplicons, we observed pronounced increases in H3K4me3 levels in plants from cross #1 and smaller increases in plants from line #3 (Fig. 3F). By contrast, H3K4me3 levels in sector *a*, which shows only a small increase in crossover frequency, were not significantly different from wild type (fig. S2B). These results suggest that the altered crossover topology within the *Coco* hotspot is directly associated with changes in the local pattern of H3K4me3 across this recombination hotspot.

To further investigate the nature of the changes observed in JMJ14-targeted lines, we compared them with changes at the *ChP* interval in lines carrying a deletion of the *Coco* region. For this, we used our previously published data from the Col-*ChP* × L*er*Δ#24 cross (*26*), normalizing the crossover distribution to that of the Col-*ChP* × L*er* wild type (Fig. 3G). The L*er*Δ#24 deletion lies within the same region as the targeted area, although it is slightly smaller than the region covered by the four gRNAs used for JMJ14 targeting (Fig. 3G). Despite this, none of the sectors within *Coco* exhibited an increase in crossover frequency relative to the wild type in the Col-*ChP* × L*er*Δ#24 cross. Consequently, the overall reduction in crossover frequency within *Coco* for this cross was greater than that observed in the Col-*ChP* JMJ14 × L*er* cross (80.3% versus 47.2%; Fig. 3H). In contrast, the other two hotspots showed virtually no reduction in crossover frequency, which differs markedly from the JMJ14-targeted lines (Fig. 3, E and H, and fig. S2A).

In summary, targeting JMJ14 to a recombination hotspot leads to a local reduction in crossover frequency in sectors directly targeted by the gRNAs. However, the hotspot is not entirely silenced, as a fraction of DSBs is still repaired via crossover in the nontargeted sectors. At the same time, the inhibitory effect of JMJ14 is also visible in neighboring, nontargeted hotspots, suggesting a local spreading of the effector. On the basis of our current data, this reduction in crossover frequency could reflect either a local decrease in SPO11-induced DSB formation or a shift in the crossover/noncrossover outcome of DSB repair, and we cannot distinguish between these possibilities.

### Targeting JMJ14 to other chromosomal regions reduces their local crossover frequency

**Fig. 4.**
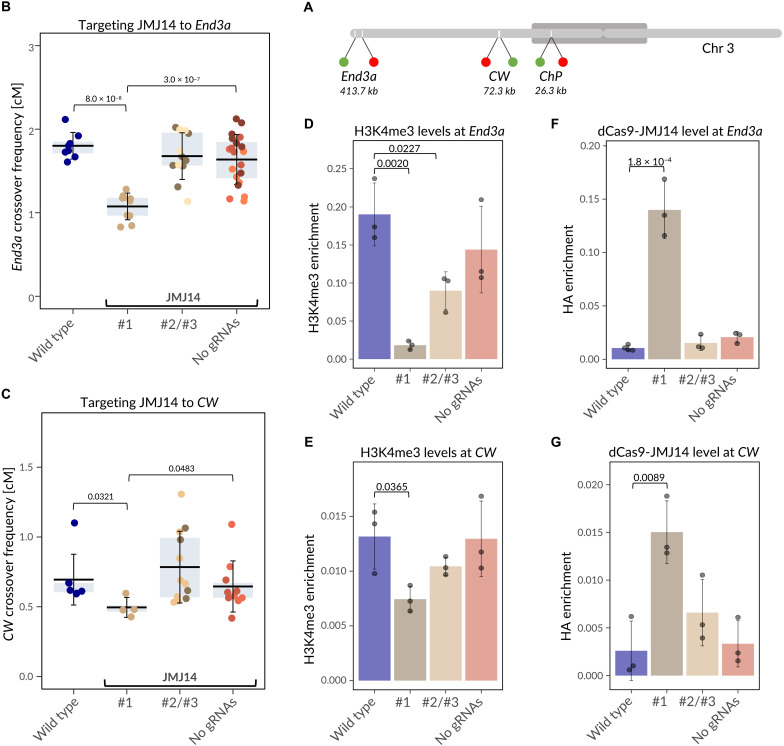
Targeting JMJ14 reduces crossover hotspot activity independently of chromosomal location. (**A**) Schematic of *A. thaliana* chromosome 3, highlighting the *End3a*, *CW*, and *ChP* intervals. Red and green markers indicate the positions of dsRed and eGFP reporters, while the dark gray oval represents the pericentromeric region. (**B**) Crossover frequency in the subtelomeric *End3a* interval for crosses with transgenic lines expressing dCas9-JMJ14 targeted to the *3a* hotspot (#1 to #3). Lines #1 to #3 correspond to independent T_1_ transformants; for lines #2 and #3, data from two independent T_1_ plants were pooled (#2/#3), because individual biological replicates (single-cross measurements for a given T_1_) showed substantial variability. Individual T_1_ lines are distinguished by different colors. Controls include wild-type Col-*End3a* × L*er* crosses and transgenic lines expressing dCas9-JMJ14 without gRNAs. In the boxplot, the center line represents the mean, the box spans the interquartile range (25th to 75th percentile), and whiskers indicate the SD. Each data point represents the crossover frequency of a single cross. Statistical significance was assessed using Welch’s test. (**C**) As in (B), but for the interstitial *CW* interval. (**D**) Changes in H3K4me3 enrichment at the nucleosome nearest to the targeted region within the *End3a* interval in the crosses used in (B), measured by ChIP-qPCR. Bars represent mean values of three biological replicates (dots), with error bars indicating SD. Statistical significance was assessed using *t* test. Only significant values are shown. (**E**) As in (D), but for the interstitial *CW* interval. (**F**) dCas9-JMJ14 enrichment at the targeted region within the *End3a* interval in the crosses used in (B), measured by ChIP-qPCR. Bars represent mean values of three to four biological replicates (dots), with error bars indicating SD. Statistical significance was assessed using *t* test. Only significant values are shown. (**G**) As in (F), but for the interstitial *CW* interval.

Conclusions drawn from a single genomic interval are subject to considerable uncertainty, particularly that the *ChP* interval is located relatively close to the centromere, and the *Coco* hotspot is one of the strongest recombination hotspots in *A. thaliana*, making it unique. To assess the broader applicability of the CRISPR-dCas9-JMJ14 system for reducing meiotic recombination via targeting H3K4me3 demethylation, we extended our approach to two additional genomic intervals. Specifically, we generated two additional reporter lines: *End3a*, a 413.7-kb interval located in a subtelomeric region containing the previously described hotspot *3a* (*13*, *30*), and *Crimson Wave* (*CW*), a 72.3-kb interval located in the middle of a chromosome arm (Fig. 4A and figs. S3 and S4). Details on the construction of *End3a* and *CW* lines are provided in Materials and Methods and fig. S5.

As in previous experiments, after transforming plants with the construct, individuals carrying the transgene were crossed with wild-type L*er* plants to measure crossover frequency based on reporter segregation in F_2_ seeds. In the *End3a* line, one of the three analyzed T_1_ lines exhibited a significant, nearly twofold reduction in crossover frequency compared to wild type (Fig. 4B; *P* = 7.95 × 10^−8^, Welch’s test). Similarly, in the *CW* line, one of three analyzed lines showed a significant reduction in crossover frequency (Fig. 4C; *P* = 0.0321, Welch’s test).

To test whether the observed decreases in crossover frequency were caused by reduced H3K4me3 levels, we analyzed this modification at nucleosomes nearest to the targeted regions. At the *End3a* interval, we detected a substantial reduction in H3K4me3 in the line showing decreased crossover frequency, down to only 0.1 of the wild-type level (*P* = 0.002, *t* test; Fig. 4D). In pooled samples #2 and #3, we also observed a moderate reduction in H3K4me3 enrichment, although these lines did not display any detectable change in recombination. Similarly, at the *CW* interval, we observed a significant decrease in H3K4me3 in the line with reduced crossover frequency, although the effect was smaller—down to 0.56 of the wild-type level (*P* = 0.0365; Fig. 4E). Notably, the reduction in crossover frequency in this line was also relatively modest (Fig. 4C). For the remaining lines that did not show changes in crossover frequency, as well as for the dCas9-JMJ14 control line lacking gRNAs, we did not detect any significant changes in H3K4me3 levels. For both intervals, the line with reduced crossover frequency showed strong enrichment of dCas9-JMJ14 at the targeted region (Fig. 4, F and G), supporting the conclusion that the changes in H3K4me3 are induced by our approach.

These observations indicate that targeting JMJ14 using a CRISPR-dCas9–based system can locally reduce meiotic recombination at any chosen hotspot, regardless of its chromosomal location. However, not all transgenic lines carrying the dCas9 construct were equally effective. Such variability is a common characteristic of genetic constructs integrated into the host genome via *Agrobacterium*-mediated transformation and is generally attributed to insertion site effects and construct expression levels (*31*). In addition, the observed reduction in crossover frequency in *no gRNA* lines suggests that, beyond the localized effect achieved through precise CRISPR-gRNA targeting, a portion of the dCas9-JMJ14 fusion protein may act nonspecifically at random genomic locations.

### Local transcriptional activity and associated chromatin changes induced by VP64 can stimulate crossover recombination

Modulating H3K4me3 levels by targeting JMJ14 resulted in both a decrease in crossover frequency and a reduction in the expression of the gene encoding *CocoRNA*. Therefore, we asked whether local induction of gene expression could increase crossover frequency in this interval. To address this, we decided to use the synthetic activation domain VP64, a derivative of the herpes simplex virus 1 (HSV-1) activation domain (*32*, *33*), targeted to the *CocoRNA* promoter via translational fusion with dCas9. VP64 is a universal transcriptional activator that functions in both animals and plants (*34*–*36*). It interacts with transcriptional mediators and RNA polymerase II, enhancing transcription initiation when delivered near the TSS (*37*, *38*).

**Fig. 5.**
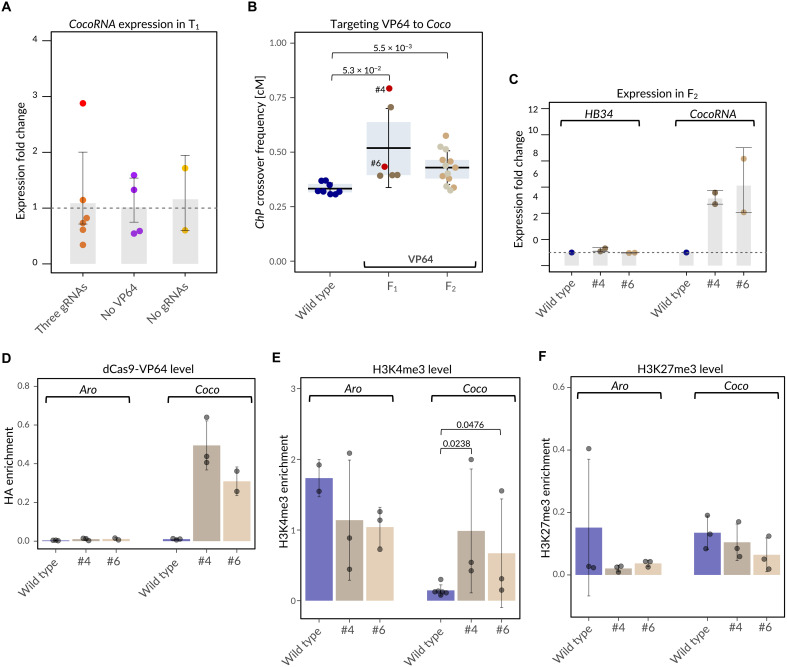
Targeting VP64 enhances crossover hotspot activity. (**A**) Relative expression of the *CocoRNA* gene within the *Coco* hotspot in T_1_ plants carrying the dCas9-VP64 construct targeted to the 5′UTR of *CocoRNA*, measured by RT-qPCR. Bars represent the mean values of biological replicates (dots), with error bars indicating SD. Each dot represents an individual T_1_ plant. A single plant exhibiting elevated expression is highlighted in red. (**B**) Crossover frequency in the *ChP* interval in crosses with the T_1_ plant exhibiting elevated CocoRNA expression [highlighted in red in (A)] in the F_1_ cross and F_2_ generation. Each data point represents a measurement for an individual plant. Offspring from F_1_ individuals #4 and #6 are shown in a single graph, distinguished by brown and gray colors, respectively (F_2_). Statistical significance was assessed using Welch’s test. (**C**) Expression levels of *HB34* (*Aro* hotspot) and *CocoRNA* (*Coco* hotspot) in F_2_ plants, measured by RT-qPCR. Bars represent the mean values of two biological replicates (dots), with error bars indicating SD. Dashed line indicates the wild type level. (**D**) Enrichment specificity of dCas9-VP64 at the *Aro* (nontargeted) and *Coco* (targeted) hotspots in F_2_ offspring from plants #4 and #6, measured by ChIP-qPCR. Bars represent the mean values of biological replicates (dots), with error bars indicating SD. (**E**) Changes in H3K4me3 enrichment at the *Aro* (nontargeted) and *Coco* (targeted) hotspots in F_2_ offspring from plants #4 and #6, measured by ChIP-qPCR. Bars represent the mean values of biological replicates (dots), with error bars indicating SD. Statistical significance was assessed using Mann-Whitney *U* test. (**F**) As for (E), but for H3K27me3 enrichment.

Since *CocoRNA* encodes a lncRNA that had not been extensively characterized, we first sought to identify its TSS using 5′ rapid amplification of cDNA ends (5′RACE) in Col and L*er*. The TSS was identical in both accessions (fig. S6), and on the basis of this information, we designed three gRNAs located +30, +234, and +477 bp upstream of the TSS. We then generated transformants expressing dCas9-VP64 targeted by these three gRNAs in the Col-*ChP* background (fig. S7A). Despite high dCas9-VP64 expression in all T_1_ lines (fig. S7B), only one line exhibited a threefold increase in *CocoRNA* expression levels (Fig. 5A). This single line was used for crosses with wild-type Col to measure local recombination rates. In the F_1_ generation, we observed an increase in *ChP* crossover frequency from 0.33 centimorgans (cM) in wild type to 0.52 cM (Fig. 5B; *P* = 6.7 × 10^−4^, Mann-Whitney *U* test).

Since these transformants were crossed with plants in Col background, self-fertilization was possible, allowing us to directly analyze the F_2_ generation. We selected progeny from two F_1_ plants differing in crossover frequency. RT-qPCR confirmed that both families of F_2_ plants exhibited similarly elevated *CocoRNA* expression compared to wild type (Fig. 5C). Expression of *HB34*, a gene located in the adjacent *Aro* hotspot, remained unchanged, indicating high specificity of transcriptional activation by dCas9-VP64 (Fig. 5C). This was consistent with strong and *Coco*-specific enrichment of dCas9-VP64 (Fig. 5D).

Furthermore, ChIP-qPCR analysis showed that F_2_ plants exhibited nearly up to threefold increase in H3K4me3 levels in the *Coco* region compared to wild type (Fig. 5E; *P* < 0.048, Mann-Whitney *U* test). As expected, no enrichment was observed for H3K27me3, a repressive chromatin mark (Fig. 5F). Crossover frequency in the *ChP* interval in the F_2_ generation was significantly higher than in wild-type plants. However, unlike in the F_1_ generation, no difference was observed between the two F_2_ lines (Fig. 5B).

In summary, targeting VP64 to the *Coco* recombination hotspot leads to chromatin modifications associated with open chromatin structure, transcriptional activation of the gene located within the hotspot, and a local increase in crossover frequency.

## DISCUSSION

Efforts to directly modulate recombination hotspot activity in plants are substantially limited by the inherently low crossover frequency and the presence of a vast number of relatively weak hotspots. A single chromosome may contain thousands of promoters and gene terminators, each of which can serve as a potential recombination hotspot (*13*). However, only one to three crossover events typically occur per chromosome during a single meiosis. Thus, detecting changes in the activity of individual hotspots requires highly specialized approaches. In this study, we used a recently developed reporter system that enables the measurement of recombination within short chromosomal intervals containing only a few recombination hotspots. By analyzing reporter segregation across thousands of seeds, we could detect subtle changes in crossover frequency within a defined interval, thereby assessing the local impact of targeted chromatin modifications on individual hotspots.

We aimed to test whether targeted chromatin regulators can locally alter meiotic recombination. To do this, we used dCas9 fused to effector proteins and guided by gRNAs. We tested the H3K4me2/3 demethylase JMJ14 and the transcriptional activator VP64 (*28*, *29*, *34*, *35*). Targeting JMJ14 to the *Coco* hotspot led to up to a twofold reduction in crossover frequency within the whole ChP interval (Fig. 2B), despite targeting being confined only to the central region of the hotspot (Fig. 1B). These changes were associated with a reduction in H3K4me3 enrichment and decreased expression of *CocoRNA*, a lncRNA gene located at the center of the *Coco* hotspot (Fig. 2, D and E). The neighboring *Aro* hotspot gene *HB34* exhibited increased transcription, suggesting coregulation of these loci (Fig. 2E). It is worth noting that JMJ14 was recently identified as one of the few effector proteins capable of inducing targeted gene repression, supporting its utility in chromatin remodeling applications (*39*).

JMJ14 targeting was carried out using four gRNAs directed to the hyperactive central region of *Coco*, the strongest hotspot within the *ChP* interval. By sequencing 164 recombinants through a seed typing approach, we were able to precisely assess the effects of JMJ14. Beyond the reduction in crossover frequency in the targeted region, we observed increases in recombination in neighboring regions of the same hotspot (Fig. 3D). This pattern contrasts with results from lines carrying a deletion of the hyperactive region of *Coco*, where a reduction in crossover frequency was not accompanied by compensatory increases elsewhere in the hotspot (Fig. 3G). These results indicate that JMJ14-mediated demethylation of H3K4me2/3 does not fully suppress crossover formation but instead leads to a partial redistribution of crossovers within the hotspot. Consistent with this, local increases in crossover frequency within a specific sector *c* of *Coco* coincide with elevated H3K4me3 levels (Fig. 3F) despite reduced *CocoRNA* transcription in JMJ14-targeted lines, suggesting that chromatin changes can promote recombination independently of transcriptional activation.

We also observed a reduction in crossover frequency at the adjacent *Aro* and *Nala* hotspots in lines with JMJ14 targeted to *Coco*, likely contributing to the overall reduction in recombination within the *ChP* interval. This effect was absent in the deletion line, in which the flanking hotspots remained unaffected. These results imply that JMJ14-mediated demethylation spreads beyond the directly targeted regions—a phenomenon commonly associated with dCas9-based systems (*40*).

Since JMJ14 targeting also reduced the expression of the targeted gene, we explored the use of a general transcriptional activator, VP64, to enhance hotspot activity. This approach proved successful: In crosses involving T_1_ lines expressing dCas9-VP64 and showing elevated expression of the targeted gene, we observed a significant increase in crossover frequency within the *ChP* interval (Fig. 5). VP64 is known to recruit histone acetyltransferases and transcriptional coactivator complexes, simultaneously affecting chromatin structure and transcription (*41*–*43*). At present, we cannot determine to what extent the increased hotspot activity is driven by enhanced *CocoRNA* transcription versus VP64-associated chromatin modifications, and these mechanisms should be considered alternative but nonexclusive. To our knowledge, VP64 variants that selectively disrupt transcriptional activation while preserving effects on H3K4me3 (or vice versa) are not available, which now precludes a clean experimental separation of these two contributions.

In summary, our experiments demonstrated that modifications of H3K4me2/3 affect meiotic recombination and can be used for local, targeted changes in crossover frequency via dCas9-mediated targeting. We also showed that epigenetic factors promoting transcriptional activation can simultaneously lead to an increase in crossover frequency. On the basis of our current data, we cannot disentangle the relative contributions of transcriptional activation and associated chromatin changes (such as H3K4me3) to these effects, and we therefore consider them as alternative, nonexclusive mechanisms. Nevertheless, the sector-level remodeling we observe—particularly the increase in crossovers within sector *c* despite reduced CocoRNA transcription (Fig. 3, D and F)—supports the idea that chromatin state can promote recombination independently of transcriptional activation and may represent the dominant proximate determinant of hotspot activity in this system. At the same time, it appears that the chromatin changes we induced do not fully explain the differences in the activity of particular recombination hotspots.

## MATERIALS AND METHODS

### Growth conditions and plant material

Plants were cultivated under controlled conditions, including a temperature of 22°C, 60 to 70% relative humidity, 150-μmol light intensity, and a 16-hour light/8-hour dark photoperiod. Seeds were stratified at 4°C for 48 hours before transferring to growth chambers.

Fluorescence-tagged lines (FTLs) used in this study included *End3a*, *Crimson Wave* (*CW*), and *Chili Pepper* (*ChP*). While *ChP* had been previously characterized (*26*), the *End3a* and *CW* lines were generated by crossing two single-color lines carrying coding sequences of the seed-expressed fluorescent transgenes (eGFP or dsRed). The single-color FTLs were provided by S. Poethig. Specifically, *CG821* and *CR921* were crossed to generate *End3a*, and *CR729* and *CG739* were crossed to obtain the *CW* interval. The exact locations and sizes of the intervals are shown in fig. S5B. F_1_ progeny were self-fertilized, and F_2_ seeds were screened using a Zeiss Lumar V12 epifluorescent stereomicroscope. Crossover events between reporter genes were identified on the basis of fluorescence patterns, specifically seeds displaying a homozygous state for one reporter and a hemizygous state for the other (GR/-R or GR/G-) were preselected. These F_2_ plants were subsequently selfed, and intervals with homozygous fluorescent reporters were selected for further study.

### RF measurements

Recombination frequencies in the obtained FTLs were assessed using a previously described seed-based fluorescence system (*26*). Seeds were collected from hemizygous *End3a*, *CW*, or *ChP* (RG/--) plants and cleaned via sieve filtration. Images of F_2_ seeds were acquired using a Zeiss epifluorescent stereomicroscope, capturing brightfield and fluorescence images through red and green filters. Seed counts were performed using a custom in-house Python script. Because of the low recombination frequencies, particularly in the *CW* and *ChP* lines, single-color recombinant seeds were manually identified. *RF*, expressed in centimorgans, was calculated using the formula: *RF* = 100 × {1 − [1 − 2(*NG* + *NR*)/*NT*]/2} where *NG* is the number of green-only seeds, *NR* is the number of red-only seeds, and *NT* is the total number of seeds collected per plant. Raw seed scoring data for all measurements are provided in data file S1.

### Preparation of sgRNA expression cassettes

The single guide RNA (sgRNA) expression cassettes were prepared as previously described (*44*). gRNAs targeting hotspots within *ChP*, *End3a*, or *CW* intervals were designed using an available tool, CRISPOR (http://crispor.tefor.net). All gRNA spacer sequences and their corresponding genomic coordinates are listed in tables S1 to S3.

### Construct preparation and plant transformation

Expression cassettes were amplified with CloneAmp high fidelity polymerase (Takara). JMJ14 histone demethylase domain was amplified from cDNA, while VP64 and dCas9-HA coding sequences were obtained from a previously described SunTag plasmid (*45*). All primers used for cloning are provided in table S4. sgRNA expression cassettes were cloned following the method described in (*46*). Genetic constructs were assembled using the Gibson assembly method (ClonExpress MultiS One Step Cloning Kit II, Vazyme), with effector domains inserted in-frame between the dCas9 and HA tag sequences.

Prepared binary constructs were introduced into *Agrobacterium tumefaciens* cells and used for plant transformation via floral dipping as described (*44*). T_1_ transformants were selected either by applying glufosinate (BASTA) or by genotyping for construct presence (primers listed in table S4).

### Chromatin immunoprecipitation–quantitative polymerase chain reaction

Chromatin was isolated from 2 g of *Arabidopsis* leaves with use of Honda buffer [440 mM sucrose, 25 mM tris-HCl (pH 8.0), 10 mM MgCl_2_, 0.5% Triton X-100, 10 mM β-mercaptoethanol, Roche Protein Inhibitor Cocktail, 10 mM phenylmethylsulfonyl fluoride (PMSF), and 40 mM spermine]. Chromatin was precleared for 1 hour with Dynabeads Protein A (Thermo Fisher Scientific), and then immunoprecipitation was carried out through overnight incubation in 4°C with 2 to 3 ng of selected antibodies (α-HA C29F4 Cell Signaling Technology, α-H3K4me3 ab8580, α-H3K4me3 04-745 Merck, α-H3K27me3 ab6002 Abcam, and α-H3 ab1791 Abcam). To capture immunocomplexes, Dynabeads Protein A were added and incubated for 1 hour in 4°C. Then, beads were washed twice with low-salt (150 mM NaCl), high-salt (500 mM NaCl), LiCl [10 mM tris-HCl (pH 8.0), 1 mM EDTA, 0.25 M LiCl, 1% NP-40, 0.5% sodium deoxycholate, and 1 mM PMSF], and Tris-EDTA (TE) buffers. Lastly, complexes were eluted by incubation with 10% Chelex in 99°C. Following treatment with proteinase K, the resulting ChIP-DNA was diluted and used as a template in qPCR reactions. Percent of input for each tested histone modifications was normalized to Input DNA.

### Reverse transcription qPCR

Thirty milligrams of unopened flower buds was collected and used to isolate RNA with the use of the RNeasy Plant Mini Kit (QIAGEN). cDNA synthesis was performed using 1 μg of total RNA and oligo-dT primers (HiScript III, Vazyme). cDNA was diluted fivefold and used as a template in RT-qPCR reactions (SYBR Green qPCR Master Mix, Thermo Fisher Scientific). Two reference genes (*AT3G18780* and *AT1G14400*) were used as controls.

### 5′ Rapid amplification of cDNA ends

The RNA from ~30-mg unopened flower was isolated with the RNeasy Plant Mini Kit (QIAGEN). One microgram of RNA was used to generate cDNA with oligo-dT and random hexamers (HiScript III, Vazyme). A 5′RACE kit (Roche) was used to capture the upstream regions of lncRNA by subsequent nested PCR reactions. Obtained amplicons were purified and cloned into pJET1.2 (Thermo Fisher Scientific) plasmid which was later transformed into competent DH5α *Escherichia coli* strain. Colony PCR with insert-specific and flanking primers was used to screen for clones with specific insert. Eight independent plasmid clones were sequenced for both Col and L*er*.

### Seed typing for the *ChP* interval

The seed typing method for fine-scale mapping of crossover breakpoints was previously described in (*26*). Briefly, recombinant seeds were manually preselected from F_2_ *Col-ChP* × *Ler* plants carrying dCas9-JMJ14 targeted to the *Coco* hotspot. DNA was extracted using a cetyltrimethylammonium bromide (CTAB) buffer and purified with AMPure XP magnetic beads (Beckman Coulter) to ensure high-quality templates essential for LR-PCR. LR-PCR was performed using PrimeSTAR GXL Polymerase (Takara Bio) under the following conditions: 0.2 to 10 ng of template DNA, 1.2 μl of 2.5 mM primers, 3 μl of buffer, 1.2 μl of deoxynucleotide triphosphates, 0.3 μl of polymerase, and distilled water to a final volume of 15 μl. The cycling conditions were 30 cycles at 98°C for 10 s and 68°C for 10 min. The *ChP* interval was amplified in three separate reactions, generating 9- to 11-kb amplicons, which were visualized by gel electrophoresis.

Amplicons from the same recombinant sample were pooled and purified with magnetic beads for library preparation, performed as previously described (*26*). Briefly, 1 μl of pooled PCR products was tagmented with tagmentation buffer [40 mM tris-HCl (pH 7.5) and 40 mM MgCl_2_], 0.5 μl of *N*,*N*′-dimethylformamide (Sigma-Aldrich), 2.35 μl of nuclease-free water (Thermo Fisher Scientific), and 0.05 μl of in-house loaded Tn5 transposase. The reaction was incubated at 55°C for 2 min and stopped with 1 μl of 0.1% SDS, followed by a 10-min incubation at 65°C. Tagmented DNA was then amplified using the KAPA2G Robust PCR kit (Sigma-Aldrich) with unique P5/P7 indexing primers (*47*). Indexed libraries were pooled and sequenced on an Illumina NovaSeq platform. Crossover breakpoints were identified on the basis of SNPs differentiating the two parental accessions.

Reads from libraries prepared from 164 recombinants preselected from F_2_ Col-*ChP* × *Ler* plants carrying dCas9-JMJ14 targeted to the *Coco* hotspot were aligned to genomic sequence of *ChP* interval with bwa-mem (*48*). Resulting bam files were sorted and indexed with samtools (*49*). Polymorphic sites were called against the previously generated high-fidelity SNP list obtained from seed typing of 243 individual F_2_ Col-*ChP* × L*er* plants (*26*). SNPs with associated reads were used to call haplotype blocks and denominate crossover sites across the *ChP* interval. Data were visualized using a publicly available custom R script deposited on Zenodo (*50*). Raw sequencing data of 164 recombinants preselected from Col-*ChP* × *Ler* plants carrying dCas9-JMJ14 targeted to the *Coco* hotspot were deposited under BioProject number PRJNA1254937.
