# Supplementary material for: Modifying meiotic recombination by targeting chromatin regulators to crossover hotspots in Arabidopsis
Source: Sci Adv. 2026 Mar 13;12(11):eaeb2890. doi: 10.1126/sciadv.aeb2890 (PMC12985737; doi:10.1126/sciadv.aeb2890)
Supplement: Supplementary file 1 — Figs. S1 to S7 Tables S1 to S4 Legend for data file S1 [file sciadv.aeb2890_sm.pdf]

Science Advances
AAAS

Supplementary Materials for

Modifying meiotic recombination by targeting chromatin regulators to crossover hotspots in *Arabidopsis*

Maja Szymanska-Lejman *et al.*

Corresponding author: Piotr A. Ziolkowski, pzio@amu.edu.pl

Sci. Adv. **12**, eaeb2890 (2026)
DOI: 10.1126/sciadv.aeb2890

The PDF file includes:

Figs. S1 to S7
Tables S1 to S4
Legend for data file S1

Other Supplementary Material for this manuscript includes the following:

Data file S1

Fig. S1.

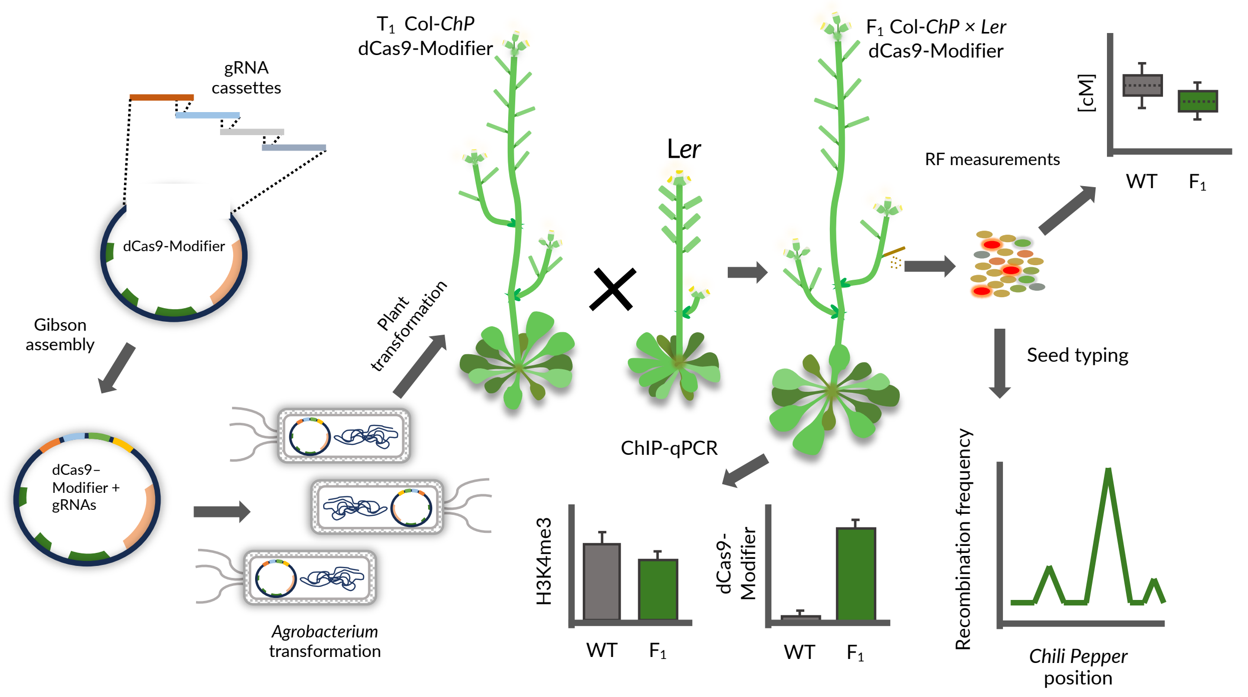

Experimental pipeline for screening H3 modifications affecting local crossover frequency. gRNAs targeting the *ChP* hotspot were cloned into a vector containing a translational fusion of dCas9 with a selected chromatin modifier and subsequently introduced into Col-*ChP* plants via *Agrobacterium*-mediated transformation. Alternatively, gRNAs targeting other hotspots were used to transform Col-*ChP*, Col-*End3a*, or Col-*CW* plants (not shown). The resulting transformants were crossed with L*er*, and the F_1 hybrids were self-pollinated. The harvested seeds were used to measure recombination frequency (RF). If a change in RF was observed compared to wild-type control plants or plants transformed with constructs lacking gRNAs (not shown), H3K4me3 levels and dCas9 binding were analyzed by ChIP-qPCR. In the case of the dCas9-JMJ14 line targeted to Col-*ChP*, a high-resolution crossover analysis was performed using the seed-typing technique.

Fig. S2.

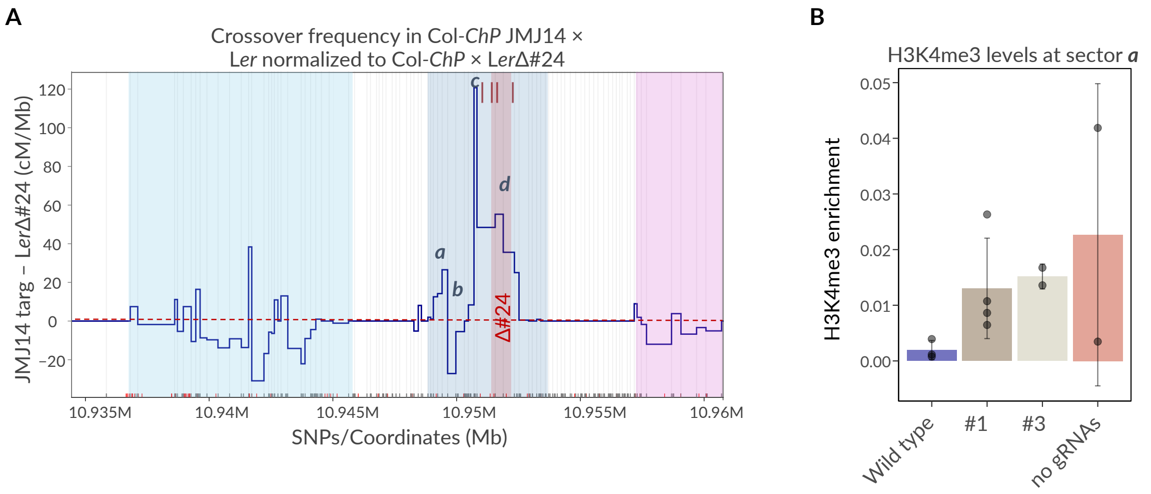

Crossover Recombination in Col-*ChP* JMJ14. (**A**) Topological changes in crossover frequency across *ChP* caused by JMJ14 targeting to *Coco*, plotted as differences from the Col-*ChP* × *Ler*Δ#24 cross (data from *ref. 23*). *Aro*, *Coco*, and *Nala* hotspots are highlighted with colored rectangles. The dashed red horizontal line indicates the Col-*ChP* × *Ler*Δ#24 crossover level. gRNAs for JMJ14 are marked with burgundy lines. Four sectors within *Coco* showing differential crossover remodeling are labeled *a*–*d*. (**B**) H3K4me3 enrichment in the '*a*' sector of the *Coco* hotspot (see (**A**)) in the crosses analyzed in **Fig. 2B**, measured by ChIP–qPCR. Cross #1 corresponds to the cross used for seed-typing and crossover mapping in (**A**). Bars represent mean values of three to four biological replicates (dots), with error bars indicating standard deviation.

Fig. S3.

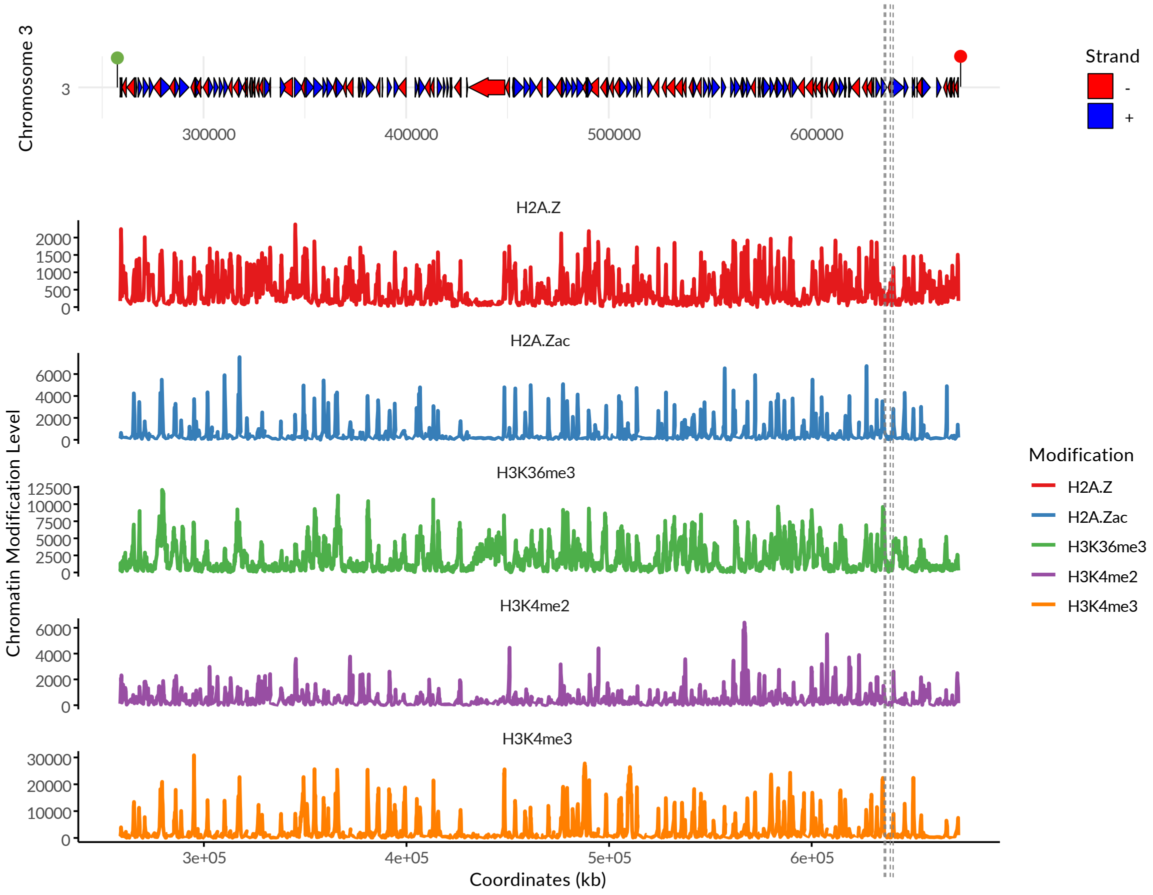

Gene structure and chromatin landscape within the *End3a* interval. Gene locations and orientations are represented by horizontal arrows (top panel). Vertical dashed lines indicate the approximate positions of the gRNAs used. Levels of specific chromatin modifications are displayed in 100 bp windows. H2A.Z and H2A.Zac levels from Ref. (*50*), H3K36me3 from Ref. (*51*), H3K4me2 from Ref. (*52*), H3K4me3 from Ref. (*53*).

Fig. S4.

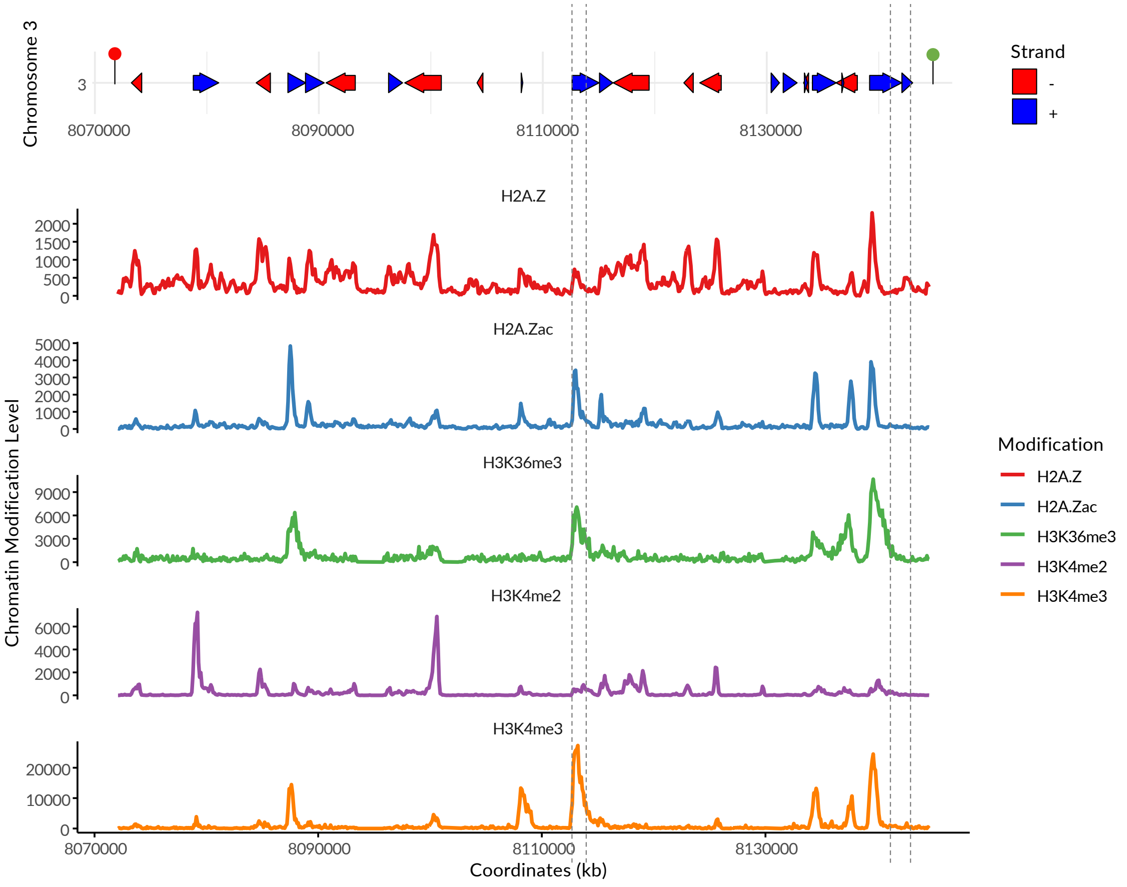

Gene structure and chromatin landscape within the *CW* interval. Gene locations and orientations are represented by horizontal arrows (top panel). Vertical dashed lines indicate the approximate positions of the gRNAs used. Levels of specific chromatin modifications are displayed in 100 bp windows. H2A.Z and H2A.Zac levels from Ref. (*50*), H3K36me3 from Ref. (*51*), H3K4me2 from Ref. (*52*), H3K4me3 from Ref. (*53*).

Fig. S5.

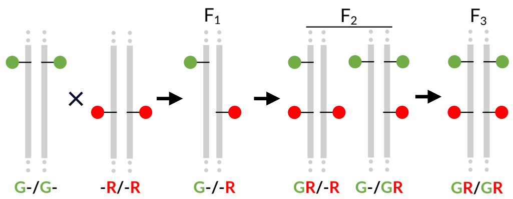

B

Interval	Size (bp)	Single-colour reporter lines	Coordinates
End3a	413,689 bp	CG821	Chr3: 258,708
		CR921	Chr3: 672,397
CW	72,294 bp	CR729	Chr3: 8,072,294
		CG739	Chr3: 8,144,588

Generation of *End3a* and *CW* lines. (**A**) Schematic overview of line generation: Plants carrying single red or green reporters were crossed, and F_2 progeny obtained by self-pollination of the F_1 generation were screened for individuals fixed for one reporter and heterozygous for the other. In the subsequent generation, plants homozygous for both reporters were selected, forming the final *End3a* and *CW* lines. (**B**) Table summarizing the key characteristics of the *End3a* and *CW* lines.

Fig. S6.

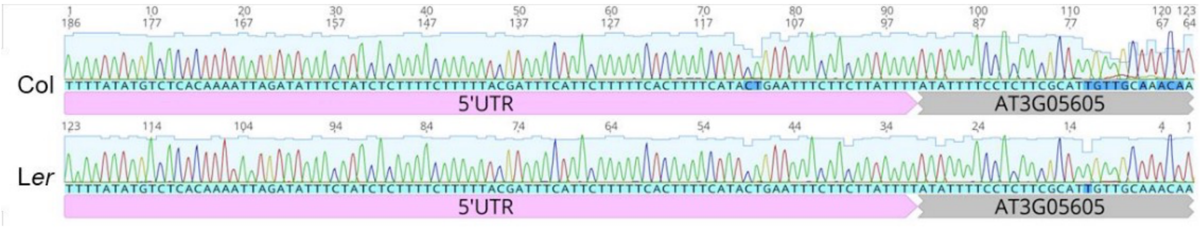

Identification of the Transcription Start Site (TSS) of the *CocoRNA* gene using 5' RACE. 5' UTR sequences of *CocoRNA* (AT3G05605) in Col (top) and L*er* (bottom).

Fig. S7.

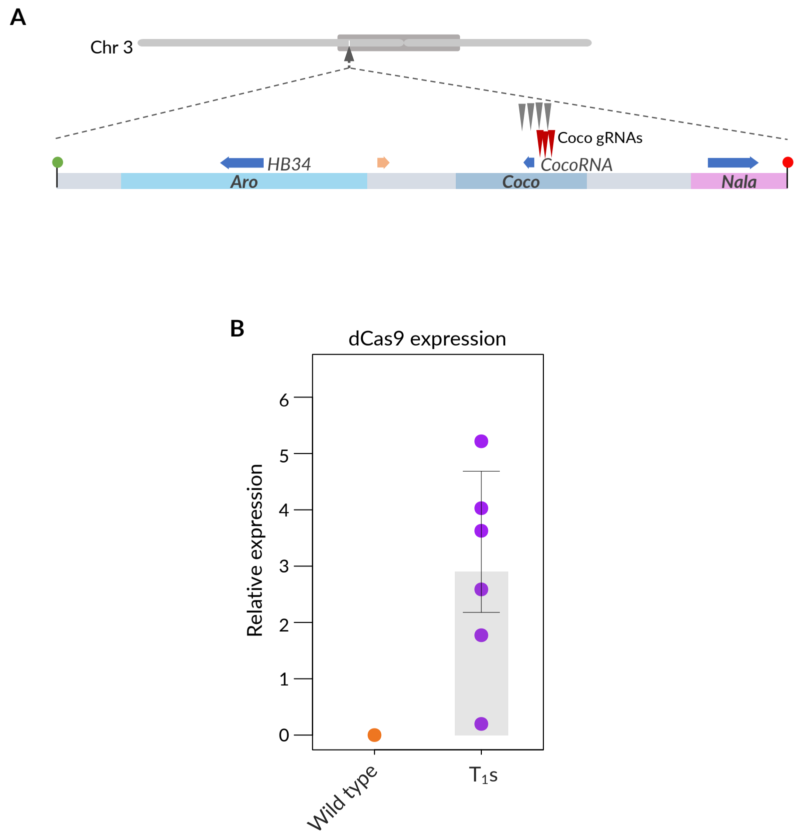

Targeting VP64 to the 5' UTR of *CocoRNA*. (**A**) Localization of gRNAs used for targeting dCas9-VP64 within the *ChP* interval. The top panel shows the position of *ChP* on the chromosome, with the pericentromeric region indicated by a dark gray oval. A zoomed-in view highlights the interval, marking the *Aro*, *Coco*, and *Nala* crossover hotspots. Blue horizontal arrows indicate gene locations, while an orange arrow marks a pseudogene. Maroon arrowheads represent gRNAs used for VP64 targeting, while gray arrowheads indicate gRNAs used for JMJ14 targeting (for comparison). (**B**) Expression levels of dCas9-VP64 in T_1 plants determined by RT-qPCR, normalized to *ACT2*. Each data point represents an individual T_1 plant (i.e., one biological replicate).

Table S1.
gRNA sequences used for JMJ14 and/or VP64 targeting to the *ChP* interval.

	**Sequence**	**Orientation**	**Coordinates (Chr. 3)**	**Col/L*er* specificity**	**Steric Hindrance**	**JMJ14 Targeting**	**VP64 Targeting**
gRNA2_Coco	ATATTAAGGCTTTAAGAGTA	Reverse	10951053..<br>10951033	Both	Yes	Yes	
gRNA6_Coco	AAATATCTTCGTCGCCAATA	Forward	10951416..<br>10951436	Both	Yes	Yes	
gRNA8_Coco	GCTTCAGTGAGAGACAAAGT	Forward	10951634..<br>10951654	Both	Yes	Yes	
gRNA9_Coco	ATTCATAATTGATCCGAG	Forward	10951884..<br>10951904	Both			Yes
gRNA10_Coco	GTACCAATTCTTGGGTGCTA	Reverse	10952284..<br>10952264	Both	Yes	Yes	Yes
gRNA11_Coco	ATTCATAAGATTCTCCATA	Forward	10952043..<br>10952063	Both			Yes

Table S2.
gRNA sequences used for JMJ14 targeting to the *End3a* interval.

	**Sequence**	**Orientation**	**Coordinates (Chr. 3)**	**Col/L*er* specificity**	**JMJ14 Targeting**
gRNA1_3a	GCTCACAAGGTACCAACCTGA	Forward	635813.. 635833	Both	Yes
gRNA2_3a	GGGCACGGTAGCCATCAATA	Forward	636515.. 636535	Both	Yes
gRNA3_3a	ATTGAGTGGTTTACCTGAACG	Forward	638921.. 638941	Both	Yes
gRNA4_3a	GCCGTGATTCTGGGGAAGT	Forward	640151.. 640171	Both	Yes

Table S3.

gRNA sequences used for JMJ14 targeting to the *CW* interval.

	Sequence	Orientation	Coordinates (Chr. 3)	Col/L*er* specificity	JMJ14 Targeting
gRNA1_CW	ATTCAAAGAGTCAGTATGATC	Forward	8113752.. 8113771	Both	Yes
gRNA2_CW	AATGATGGGCTTGATCGTTT	Forward	8112690.. 8112708	Both	Yes
gRNA3_CW	ATTGCTCTTTAGTTCTTGA	Forward	8140865.. 8140884	Both	Yes
gRNA4_CW	ATTACTCAAAAAACCGAAACG	Forward	8142639.. 8142658	Both	Yes

Table S4.
List of primers used in the study.

ID	Sequence 5'--> 3'	Comments
dCas9_F_1	TTACAATTACAAAAAAAGTTAACCCCGGGCAATTACAAAAAAAGTTAACATGGACA	Primers used to prepare the vector
dCas9_R_2	CACTTTCCGCTTTTTCTTAGGATCTTCCACCTTGCGTTTTTTCTTGGGGGAGTCGCCTCCCAGCTGAGACA	
dCas9_F_3	AAAAAGCGGAAAGTGGGATCCCGTACGGGCGGTGGCGGAGGGG	
dCas9_R_4	CCACCACCTTAACCACCACCAGCGTAATCTGGAACATCGTATGGGTAGGATCCCCCTCCGCCACCGCCACTACC	
dCas9_F_5	GTGGTTAAGGTGGTGGCTGCTTTAATGAGATATGCG	
dCas9_R_6	TATGCACAGGCCAGGCGGGTT	
dCas9_F_7	CCTGGCCTGTGCATAACTGTCTGGCCAGCGCACAG	
dCas9_R_8	ACAAGCTCGAGTTTCTCCAT	
dCas9_F_9	GAATTTTCAAATAAACCTAAGGCCCGGGAGTCAATTCATGCAGCAACCC	
dCas9_R_10	CCCGGGGTTAACTTTTTTTGTAATTGTAAATAG	
dCas9_R_11	TTTATTTGAAAATTCATAAGAAAAGC	
dCas9_F_12	GAAACTCGAGCTTGTTTTTCAAATCAGTGCGCAAG	
VRF1_dCas9	GAATTTTCAAATAAACCTAAGGGGATGGCTCGAGTTTTCAGC	Primers used for cloning the gRNAs
R1VF_dCas9	TGCATGAATTGACTCCCGGGCCTAAGGGCTGCAGGAATTCGATATCAAGC	
Construct_F	CTGAGCGCCTCTATGATCAAGA	Primers to genotype for the construct presence
Construct_R	AGCTGCATGAAGTTTCTGTTGG	
JMJ14_F	TAAGAAAAAGCGGAAAGTGGATCAGCTTGCATCTCTAGCAGAGT	Primers to amplify coding sequence of JMJ14
JMJ14_R	GAACATCGTATGGGTAGGATAGGACTTATCTCCATCTTATCAACC	
At1G71220_ChIPqF	TGAGGCTGGGTAATGCTTCT	Primers used for ChIP-qPCR or RT-qPCR
At1G71220_ChIPqR	AATCGGAATCATCTCCATCG	
Coco_MSz347_F	CGTCAACTCCAATTTCATCAACA	
Coco_MSz334_R	GGATTCATTGAGTTTACCATATTGGC	
Aro1_ChIPqF	GGACAGAGCTTGAAGATGTTTAAC	
Aro1_ChIPqR	GGACTGTCTTTAGGTTTAAGAC	
Aro2_ChIPqF	CTCATGCATTGTGACACTTCTTG	
Aro2_ChIPqR	CAAAGTTGTTCGGTAATTACATTG	
Coco1_ChIPqF	TGGTCATTCCCGTCTTAAAACTT	

Coco1_ChIPqR	GAGTACCTTATGGAGAATCTTATG	
Coco2_ChIPqF	CTTACATAGATATCCAAATGGACAC	
Coco2_ChIPqR	CCACTCAACGTTGTACTGATATCT	
Coco3_ChIPqF	GAGTGGCCCCTTTTAAAGAAC	
Coco3_ChIPqR	TGCCTCCAAGCTATATGATATAAGT	
Coco4_ChIPqF	GGAGGCATAACTTACATTTTCTAG	
Coco4_ChIPqR	CCGATTTATAAATCTACCTCGTGT	
Coco_a_ChIP_F	ACCGTTCATAAAATTTGTTTTAGTGGT	
Coco_a_ChIP_R	AAAGATTTTTCTTCTTAGGGCCAA	
Coco_c1_ChIP_F	TTTCACCAAAGCTTCCTCTGC	
Coco_c1_ChIP_F	CGTAAGTATTTAATGAACATAATTCACCG	
Coco_c2_ChIP_F	CGGTGAATTATGTTCATTAAATACTTACG	
Coco_c2_ChIP_F	TGTATTCGTTATAGTTTATATGTTCGGT	
End3a_ChIP_F	ACGGTCTTGCTCCCATCATTA	
End3a_ChIP_R	AGGACGCCAAAGCTATAGACA	
CW_ChIP_F	TGGGAAATACGTTTCAAAGAGTCA	
CW_ChIP_R	TTCGCATCAGTCATCTTCGTC	
lncRNA_RTqPCR_F	ACACTCTAAGCAAGCCACGA	
lncRNA_RTqPCR_R	GCCTCACTGTGCTAGCTTCA	
HB34_RTqPCR_F	GGCGGCGAGAACAGTAACGA	
HB34_RTqPCR_R	GCCACCATCAGCTCCTCCAC	
Cas9_RTqPCR_F	CAAGAACCTGTCCGACGCCA	
Cas9_RTqPCR_R	GAGCTTTCAGCAGGGTCAGG	
UBC_RTqPCR_F	CTGCGACTCAGGGAATCTTCTAA	
UBC_RTqPCR_R	TTGTGCCATTGAATTGAACCC	
UBQ_RTqPCR_F	GGCCTTGTATAATCCCTGATGAATAAG	
UBQ_RTqPCR_R	AAAGAGATAACAGGAACGGAAACATAG	

ACT2_RTqPCR_F	GAGAGATTCAGATGCCCAGAAGTC	
ACT2_RTqPCR_R	TGGATTCCAGCAGCTTCCA	
ChP_1_Col	TGATAGTTCTCCGAAGAATACTTCCAT	Primer used for LR-PCR (Region 1)
ChP_1_Ler	GAAGGAAGGAGACAACTCTGATACT	
ChP_1_R	ACGACCTTCTTATTTGCCAATTCAT	
ChP_2_F	GATTGGTTTAGCTGGTTGGATCCG	Primers used for LR-PCR (Region 2)
ChP_2_R	TACTTTTGCTCCTCACACCCAAGA	
ChP_3_F	AGCTTCCTCTGCCACTAAATCACA	Primers used for LR-PCR (Region 3)
ChP_3_R	TTTTCAGACAAACTCCAATTTCACAG	
GSP1	GGAATCTAAACTCTAAAATTATATATAGGG	Primers used in 5'RACE
GSP2	GCCTCACTGTGCTAGCTTCA	
GSP3	GTGTTGCAGCTTGTTTGCAA	
GSP4	TGAATAATCGTGGCTTGCTTAGA	

Supplementary Data

Data File S1 (Excel, “Source Data”).
Contains the raw numerical values underlying the plots shown in Figs. 1D,E; 2B–E; 3F; 4B,D–G; 5A–F; and Figs. S2 and S7.
